# Effects of Assisted Reproduction Technology on Placental Imprinted Gene Expression

**DOI:** 10.1155/2010/437528

**Published:** 2010-07-12

**Authors:** Yukiko Katagiri, Chizu Aoki, Yuko Tamaki-Ishihara, Yusuke Fukuda, Mamoru Kitamura, Yoichi Matsue, Akiko So, Mineto Morita

**Affiliations:** Reproduction Center, Department of Obstetrics and Gynecology, Toho University Omori Medical Center, 6-11-1 Omori-Nishi, Ota-ku, Tokyo 143-8541, Japan

## Abstract

We used placental tissue to compare the imprinted gene expression of IGF2, H19, KCNQ1OT1, and CDKN1C of singletons conceived via assisted reproduction technology (ART) with that of spontaneously conceived (SC) singletons. Of 989 singletons examined (ART *n* = 65; SC *n* = 924), neonatal weight was significantly lower (*P* < .001) in the ART group than in the SC group, but placental weight showed no significant difference. Gene expression analyzed by real-time PCR was similar for both groups with appropriate-for-date (AFD) birth weight. H19 expression was suppressed in fetal growth retardation (FGR) cases in the ART and SC groups compared with AFD cases (*P* < .02 and *P* < .05, resp.). In contrast, CDKN1C expression was suppressed in FGR cases in the ART group (*P* < .01), while KCNQ1OT1 expression was hyperexpressed in FGR cases in the SC group (*P* < .05). As imprinted gene expression patterns differed between the ART and SC groups, we speculate that ART modifies epigenetic status even though the possibilities always exist.

## 1. Introduction

Assisted reproduction technology (ART) is associated with epigenetic alterations [1–3] that can affect fetal growth in animals and humans and usually results from imprinting. Followup studies of ART-conceived children have shown that ART does not increase the incidence of congenital abnormalities [4–10]; however, it increases the incidence of epigenetic disorder diseases, such as Beckwith-Wiedemann Syndrome (BWS), Angelman Syndrome (AS), and Russell-Silver Syndrome (RSS) [11–17].

In BWS [MIM 130650] and RSS [MIM 180860], abnormal fetal growth is a major phenomenon, and abnormal prenatal development has been associated with the epigenetics of some imprinted genes. Reduced birth weight, which is occasionally observed in infants conceived by ART, is an important consideration as it is associated with adult diseases such as insulin insensitivity, polycystic ovary syndrome, and cardiovascular diseases [18–20]. Therefore, normal prenatal development may be very important not only for childhood health but also for long-term health. Here, we used human placental tissue to compare the imprinted gene expression of IGF2, H19, KCNQ1OT1, and CDKN1C genes known to be associated with fetal growth, in ART-conceived singletons with that in spontaneously conceived (SC) singletons.

## 2. Materials and Methods

A total of 1302 singletons delivered at our center from June 2005 to March 2007 were enrolled in this study. Of these 1302 potential subjects, 313 were excluded due to complications. A total of 860 infants had appropriate-for-date (AFD) birth weight (2500 g ≤ AFD birth weight < 3500 g), 64 cases exhibiting fetal growth retardation (FGR) had a birth weight of <2500 g, and 65 cases had a birth weight of ≥3500 g. Thus, 989 subjects (ART *n* = 65; SC *n* = 924) were assessed with 3 idiopathic FGR cases in the ART group and 61 in the SC group (Table 1).

For the gene expression study, placental tissue was collected from 297 cases after receiving informed consent under the IRB protocol of our center for genetic analysis (Table 2). Total RNA was extracted from the fetal placenta, and reverse transcription was performed. Gene expressions of IGF2, H19, KCNQ1OT1, and CDKN1C were analyzed by real-time PCR with GAPDH serving as the endogenous control. 

## 3. Results and Discussion

**Figure 1:**
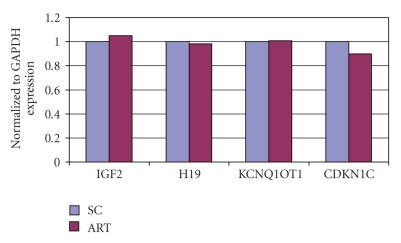
Gene expression of placental tissue. ART versus SC in AFD birth weight cases. ART: assisted reproductive technology. SC: spontaneous conception. AFD: appropriate-for-date. Results of gene expression analysis compared with the endogenous control GAPDH. In AFD birth weight cases, gene expression patterns were similar in both the ART and SC groups.

**Figure 2:**
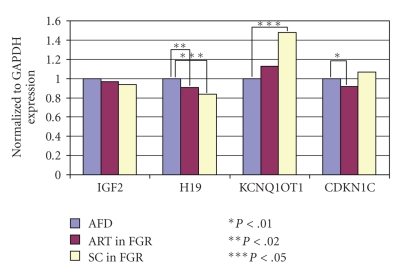
Gene expression of placental tissue. ART versus SC in FGR cases. There were no differences in the gene expression of IGF2; however, H19 expression was significantly reduced in FGR cases both in the ART and SC groups compared with the AFD birth weight cases (*P* < .02 and *P* < .05, resp.). Conversely, KCNQ1OT1 was hyperexpressed in FGR cases in the SC group (*P* < .05), while CDKN1C expression was reduced in FGR cases in the ART group (*P* < .01).

**Figure 3:**
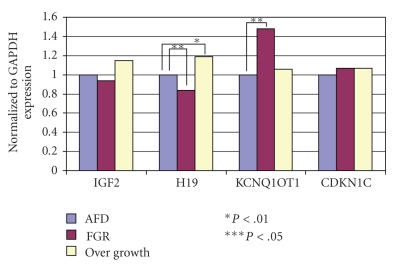
Gene expression in placental tissue. FGR and birth weight ≥3500 g cases in the SC group. H19 expression was significantly reduced in FGR cases, but significantly enhanced in cases with a birth weight of ≥3500 g (*P* < .01).

The mean birth weight was significantly lower (*P* < .001) in the ART group (2905.1 ± 459.0 g) than in the SC group (3607.9 ± 589.9 g). The mean placental weight, however, showed no significant difference (ART = 689.3 ± 152.6 g; SC = 613.0 ± 142.5 g) (Table 3). Gene expression patterns in the AFD birth weight cases were similar in both the ART and SC groups (Figure 1). H19 expression was reduced in FGR cases both in the ART and SC groups compared with the AFD cases (*P* < .02 and *P* < .05, resp.) (Figure 2). Conversely, H19 expression was significantly enhanced in SC cases with a birth weight of ≥3500 g (*P* < .01) (Figure 3). On the other hand, CDKN1C expression was reduced in ART cases with FGR (*P* < .01), and KCNQ1OT1 appeared to be hyperexpressed in SC cases with FGR (*P* <  .05) (Figure 2). The expression of other genes examined showed no difference from the control. 

The results demonstrated that birth weight was significantly lower in the ART group than in the SC group, which is in agreement with the results of other studies [21–23]. Some followup studies of ART-conceived children suggest that low birth weight is due to multiple pregnancies. However, even in singleton cases, low birth weight has been observed in infants conceived by ART. For cases conceived using fresh embryo replacement, birth weight was comparably lower than that for cases conceived using cryopreserved embryos [24, 25]. Although we did not separate cases conceived with fresh embryos and cryopreserved embryos, many cases in this study were conceived by fresh embryo replacement. On the other hand, placental weight showed no significant difference between the ART and SC groups. In other studies, however, placental thickness was significantly larger in ART cases than in SC cases, but there were no differences in morphological or histopathological features of the placenta between both groups [26]. There were no differences in the gene expression patterns in the AFD cases between the ART and SC groups. However, the expression of H19, a paternally methylated imprinted gene, was reduced in FGR cases in both the ART and SC groups. As maternally expressed genes such as H19 enhance fetal development, the hypoexpression of H19 affects fetal development. Here, we established the relationship between the hypoexpression of H19 and reduced fetal weight. Additionally, CDKN1C, another maternally expressed gene, exhibited reduced expression in FGR cases conceived by ART. In contrast, the expression of KCNQ1OT1, a paternally expressed gene with a complementary relationship to CDKN1C, was enhanced in FGR cases conceived by natural conception. In this study, we confirmed differences in the expression of imprinted genes in the placental tissue of infants conceived by ART. However, even in the SC cases, epigenetic alteration has been observed. The loss of imprinting on genes located on chromosome 11 is identified as a cause of poor fetal growth in humans [27], which is also reflected in our study. We postulate that ART could affect the epigenetic characteristics of male and female gametes or it can have an impact on early embryogenesis. Additionally, ART could be associated with an increased risk of genomic imprinting abnormalities as epigenetic reprogramming occurs during gametogenesis or immediately following fertilization [28–32].

## 4. Conclusions

Imprinted gene expression patterns of placental tissue in FGR cases were altered compared with cases of normal fetal growth. However, imprinted gene expression patterns of placental tissue in ART cases were different from those of SC cases. In cases with a birth weight of ≥3500 g, gene expression differed from cases with standard fetal growth. While we recognize the possibility of changes in epigenetic status in any pregnancy, we speculate that epigenetic status is altered by ART. Although ART has been widely accepted and safety performed, epigenetics should remain an important factor for evaluating the safe development of reproductive medicine, as well as for considering the health of the next generation.

## Figures and Tables

**Table 1:** Subject characteristics.

	ART (*n*)	SC (*n*)	Total (*n*)
AFD (2500 g ≤, <3500 g)	62	798	860
FGR (≤ 2500 g)	3	61	64
OG (≥3500 g)	—	65	65
Total	65	924	989

*n*: number of cases, AFD: appropriate-for-date, FGR: fetal growth retardation, OG: over growth, ART: assisted reproductive technology, and SC: spontaneous conception.

**Table 2:** Imprinted gene expression analysis in placental tissue samples.

	ART (*n*)	SC (*n*)	Total (*n*)
AFD (≥2500 g, <3500 g)	45	173	218
FGR (≤2500 g)	3	51	54
OG (≥3500 g)	—	25	25
Total	48	249	297

*n*: number of cases, AFD: appropriate-for-date, FGR: fetal growth retardation, OG: over growth, ART: assisted reproductive technology, and SC: spontaneous conception.

**Table 3:** Birth weight and placenta weight.

		Weight (g)	
	*n*	Neonate	Placenta
ART	65	2905.1 ± 459.0*	589.3 ± 152.6
SC	924	3607.9 ± 589.9*	613.0 ± 142.5

*P < .001. *n*: number of cases, ART: assisted reproductive technology, and SC: spontaneous conception.
